# Prevalence of Women's Home Birth Preferences and Its Associated Factors in Ethiopia: A Systematic Review and Meta-Analysis

**DOI:** 10.1155/2024/5780900

**Published:** 2024-11-06

**Authors:** Temesgen Geta, Dereje Haile, Abiy Girma

**Affiliations:** ^1^School of Nursing, Wolaita Sodo University, Wolaita, Ethiopia; ^2^School of Public Health, Wolaita Sodo University, Wolaita, Ethiopia

**Keywords:** Ethiopia, home delivery, preference, systematic and meta-analysis, women

## Abstract

**Background:** In low-income countries, such as Ethiopia, home birth is the main cause of maternal and neonatal mortality. Several separate studies have been conducted on the prevalence of home birth preference. However, there is no pooled prevalence of home birth preferences. So, this systematic review and meta-analysis is aimed at assessing the overall preference for home birth and related factors among Ethiopian women.

**Methods and Materials:** The review included only published articles. Medline/PubMed, Web Science, Google Scholar, Scopus, and the Cochrane Library are the main databases. The review includes cross-sectional studies in English that meet eligibility requirements. The combined prevalence of women's preference for home birth is calculated by random effect models. In addition, Egger's tests and funnel diagrams were used to investigate publication biases. STATA Version 14 is used to perform all statistical analyses.

**Results:** The review included 14 studies with 6631 participants. In Ethiopia, the prevalence of women's preference for home birth was 41.48% (confidence interval (CI): 49.99; 63, 56; I2:98.7%; *p* ≤ 0.001). In the analysis of the subgroups, the Oromia region had the highest home birth preference rate at 61.40% (55.54%, 67.16), while southern Ethiopia had the lowest value at 20.52% (5.18, 29.75). The probability of preferring home birth was higher for women without education (OR = 0.22, *p* ≤ 0.001, *I*^2^ = 69.7%) and for younger women (ODR = 0.47, *p* ≤ 0.001, *I*^2^ = 84.2%).

**Conclusion:** According to the study, 41% of Ethiopian women prefer home births over institutional births. Age and education of women are statistically important factors in the choice of birthplace. To solve this problem, health professionals and other stakeholders are strongly encouraged to provide women's health education at the community and institutional levels.

## 1. Background

The World Health Organization aims to improve maternal health, improve research, provide clinical and programmatic guidelines based on evidence, set global standards, and provide technical assistance to develop and implement effective policies and programs to reduce maternal mortality [1]. However, in 2020, 800 women around the world died every day due to preventable causes associated with pregnancy and birth. Seventy percent of these deaths occurred in sub-Saharan Africa [1]. Women in these countries are at greater risk of death during pregnancy than women in other regions. This can be avoided by qualified caregivers who give birth to women in health facilities [1, 2]. Delivery outside health facilities is a public health issue, common in low- and middle-income countries. Evidence indicates that 34% of women in sub-Saharan Africa are born outside health facilities, causing serious complications for mothers and babies [3–5].

The risk of newborn death at home is 21% higher than that at hospital birth. The maternal mortality rate of women who gave birth at home is three times that of women who gave birth in hospitals [6, 7]. In 37 countries in Sub-Saharan Africa and South Asia, the literature indicates that noninstitutional delivery is more likely to cause infant mortality. Premature mortality can increase by 5% if delivery occurs outside health facilities [8]. Labor, delivery, and postpartum periods are the most dangerous periods in women's and children's lives in developing countries. As a result, preferential birthplaces for pregnant women are an important aspect of women's health care [9–11]. The place of delivery is usually an important indicator of the quality of the services provided by health professionals in hospitals.

Although the Ethiopian Government has undertaken various measures to expand health facilities, hire qualified health workers, and launch health expansion programs, more than 50% of Ethiopian women still have children at home [12, 13]. In order to prevent women from having children at home, it is essential to assess potential factors affecting women's choice of birthplace and to provide the basis for implementing programs and enabling policy makers to implement programs for the transformation of the health sector [14, 15].

Factors leading to the choice of home birth include distance from home, poor quality of health care, level of education, low socioeconomic status, poor provider attitudes and client knowledge of birth complications, sociocultural beliefs, occupational status of women or partners, parity, pregnancy, age, previous prenatal care, and inaccessibility of health services [16–19]. Reviews from different countries show that women's preference for the home birthplace was 22% in Liberia [16], 24.7% in Ghana [17], Nigeria (15%) [20], 11.7% in Eretria [21], 61.2% in Gojjam [19], 58.7% in Haramaya [22], and 58.7% in Tigray [23], respectively.

Although several individual studies have been conducted to determine women's preference for home birth in different regions of Ethiopia [19, 22–34], there is no nationwide data to show overall prevalence. And the representativeness and results of a single study are neither conclusive nor consistent. Therefore, the purpose of this systematic review and meta-analysis was to assess the pooled prevalence of women's preference for home birth and associated factors in Ethiopia. The results of this study lead to general insights that help reduce the preference for home birth by helping to develop policies, design strategies, and improve the use of institutional delivery. This plays an important role in reducing maternal and neonatal mortality.

## 2. Methods and Materials

A systematic review was conducted to estimate the overall prevalence of women's preference for home birth in Ethiopia.

### 2.1. Search Strategy

Between October 1 and October 30, 2023, databases such as Medline/PubMed, the Science Web, Google Scholar, Scopus, and the Cochrane Library were used to search for research. We checked the databases of https://www.crd.york.ac.uk/prospero and the Cochrane Library to ensure that no review had been carried out before and to avoid duplication. International Prospective Systematic and Meta-Analysis Registration Register (PROSPERO) also recorded this registration with CRD42023454700. The review includes research conducted between 2013 and 2023.

A comprehensive search strategy was developed using multiple Boolean operators against standard population comparison and intervention outcome (PICO) questions. The words “OR” and “AND” were used as Boolean operators. The terms “home delivery preference” OR “home birth preference” OR “choice of home delivery” OR “choice of home birth” OR “noninstitutional delivery preference” AND “associated factors” OR “influencing factors” AND “Ethiopia” are searched using Boolean operators. All articles retrieved from the databases were checked for title and abstract before being exported to the Endnote library. These articles met the criteria for inclusion in terms of titles and abstracts that were read in full. Three authors (T.G., D.H., and A.G.) performed a search strategy. The PRISMA (Preferred Reporting Items for Systematic Review and Meta-Analysis) protocol was used to select and direct the selection of articles for this review.

### 2.2. Eligibility Criteria

#### 2.2.1. Inclusion and Exclusion Criteria

The articles eligible for this review assess women's home birth preferences in Ethiopia. The studies conducted in a cross-sectional study design and published in English were included in this study. In addition, it included participants who lived in Ethiopia. It also included studies conducted from 2013 to 2023. Studies that did not address the prevalence of women's preference for home births were excluded. This review excluded studies conducted outside of Ethiopia.

### 2.3. Data Extraction

PRISMA was used to select and select the articles for this review. The parameters used to extract data include the author's name, publication year, study area, sample size, study population, and study design. Using Microsoft Excel spreadsheets, we collected the required data from accepted articles. The three authors (T.G., D.H., and A.G.) independently extracted the information from the supplementary document. The studies that met the admission requirements were included after a detailed agreement and discussion on data extraction, and summarized in the table.

### 2.4. Assessment of Risk of Bias and Quality Assessment

To assess the quality of the study, a critical analysis was carried out with the meta-analysis and statistical evaluation tool of the Joanna Brings Institute. The Joanna Briggs evaluation tool determines whether studies and abstracts should be included in articles. The quality of the articles was evaluated prior to the final assessment. Cross-sectional studies were evaluated based on source population, sample size, data collection methods, data collection tools, statistical analysis, and response rates, and scored on a 1- to 9-point scale. A score of seven or more quality assessment indicators is considered to be a low risk of bias (Table 1).

### 2.5. Data Processing and Analysis

Microsoft Excel spreadsheets and STATA Version 14 are used to extract data and analyze data, respectively. The random effect model analysis is used to calculate the pooled prevalence of women's preference for home birth in Ethiopia. Using funnel plot and visual analysis, biases in the publication were investigated. The heterogeneity of the study was tested with Cochrane Q-Static and *I*^2^. The preference for home birth among women in the regions was compared to an estimated prevalence using a subgroup analysis. A forest plot format with a 95% CI was used to show a pooled prevalence. *p* value ≤ 0.001 is considered as the level of significance for the forest plot.

## 3. Result

### 3.1. Identification and Characteristics of Included Studies

**Figure 1:**
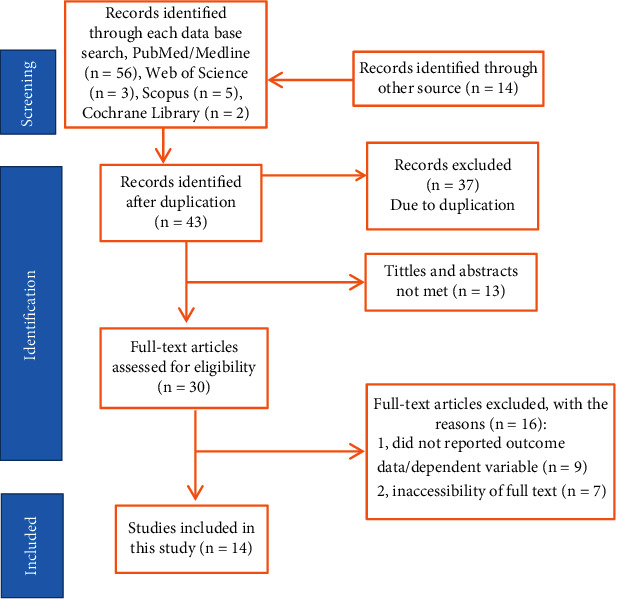
PRISMA flow diagram of study selection for the systematic review of women's choice of home birth among delivered mothers in Ethiopia, 2023 (*n* = 14).

Between October 1 and October 30, 2023, 80 articles were identified in the main electronic databases and other applicable sources were searched. Of these identified articles, 37 have been removed due to duplicates, and 43 are reserved for further review. Thirteen studies were excluded because the abstract and title did not meet the requirements. Of the remaining 30 articles, 16 were excluded due to inconsistent inclusion criteria for this review. Finally, 14 studies met the eligibility criteria for this study (Figure 1).

This systematic review and meta-analysis included a total of 14 articles with 6631 participants. All studies were cross-sectional studies, and the sample size ranged from 277 [34] to 901 [33]. The regional distribution of the study included two in the Oromia Region [22, 34], five in the Amhara region [19, 24–26, 30], three in the Southern Nations, Nationalities People's Region (SNNPR) [28, 29, 31], and one in Somalia [27], Tigray [23], Addis Ababa [33], and Sidama [32] (Table 2).

### 3.2. Prevalence of Women's Preference for Home Birth in Ethiopia

**Figure 2:**
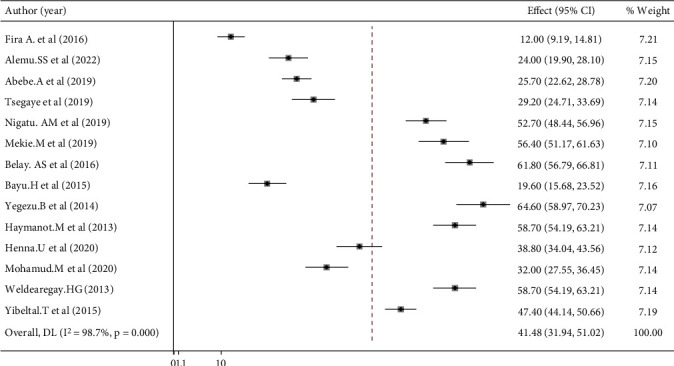
Forest plot showing the pooled prevalence of women's preference of home birth in Ethiopia (*n* = 14).

According to this review, women prefer home birth, ranging from 12% [28] to 64.6% [34]. The estimated pooled prevalence of women's home birth preferences in Ethiopia is 41.48% (95% CI (49.99; 63.56); *I*^2^ = 98.7%, *p* ≤ 0.001) (Figure 2).

### 3.3. Subgroup Analysis of Women's Preference for Home Birth in Ethiopia

**Figure 3:**
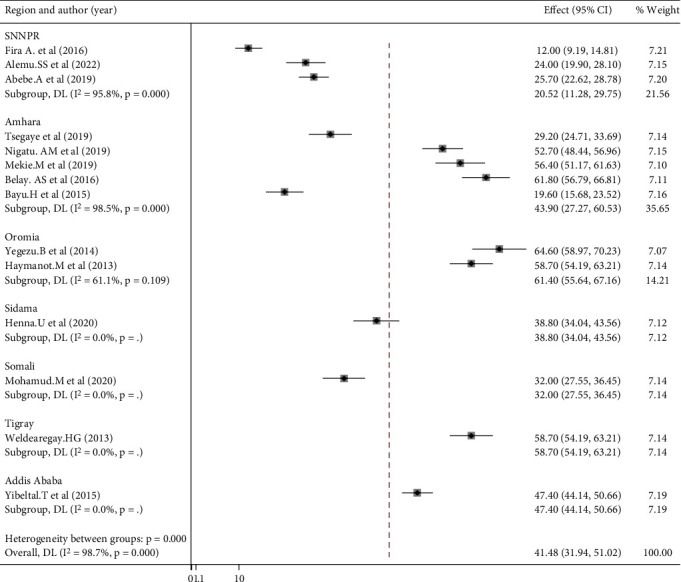
Subgroup analysis of women's preference of home birth by region in Ethiopia (*n* = 14).

Subgroup analysis performed for each region revealed that Oromia had the highest prevalence of women's home birth preference with a score of 61.40% (95% CI: 55.64, 67.16), followed by Tigray with 57.8% (95% CI: 54.19, 63.21). The lowest value was observed in the SNNPR region at 20.52% (95% CI: 11.28, 29.75). (Figure 3).

### 3.4. Heterogeneity and Publication Bias

**Figure 4:**
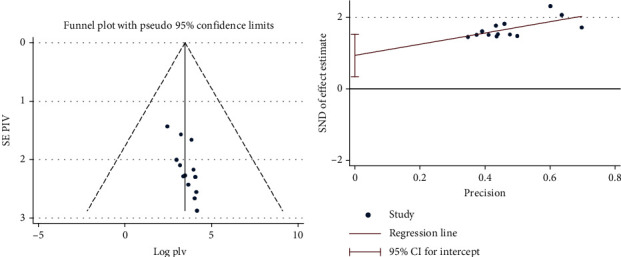
(a) Funnel plot and (b) Egger's test of the review.

To minimize and control the heterogeneity of the study, we performed a regional subgroup analysis. The results of the *I*^2^ test show that there were significant differences between the studies (*I*^2^ = 98.7% with *p* ≤ 0.001). The studies' publication biases were monitored using Egger's tests and visual inspection of a funnel plot. The results of the funnel diagram showed that the study selected had a symmetric distribution after inspection and Egger's test. This showed that there were no potential biases (Figures 4(a) and 4(b). Furthermore, we conducted sensitivity analysis by eliminating steps to assess the effects of single studies on overall effect estimates. The results show that the removal of a single study has had no significant impact on the collective prevalence (Table 3).

### 3.5. Factors Associated With Women's Preference for Home Birth in Ethiopia

**Figure 5:**
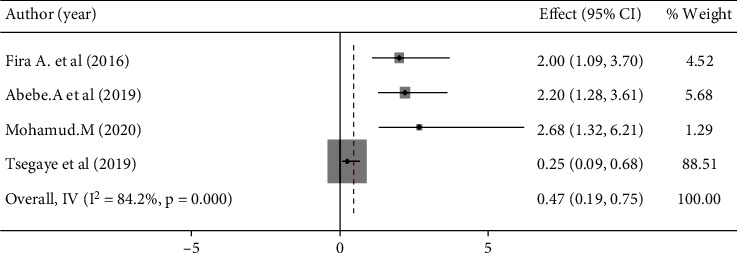
Women's educational level association with the choice of home birth in Ethiopia.

**Figure 6:**
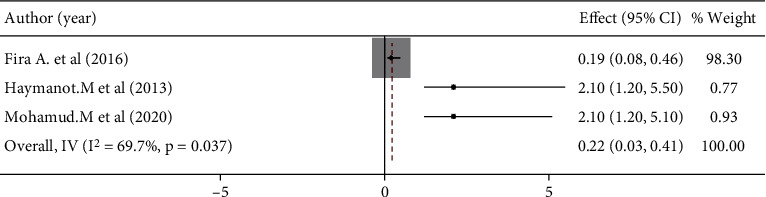
Women's age association with the choice of home delivery in Ethiopia.

**Figure 7:**
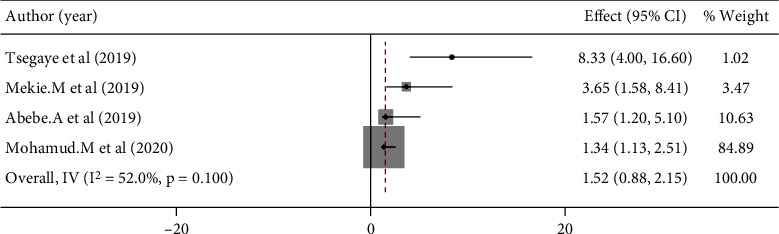
History of antenatal care follow-up association with the choice of home birth in Ethiopia.

**Figure 8:**
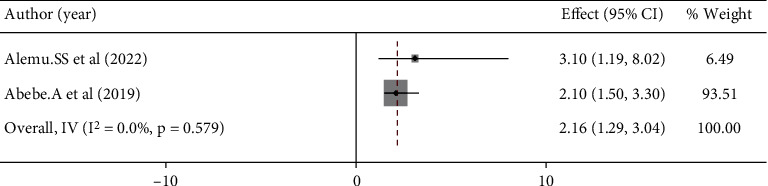
Association between women's knowledge status on labor and delivery complications and choice of home birth in Ethiopia.

Women's age and education were significantly associated with women's preference for home birth (Figures 5 and 6), but lack of prenatal care follow-up and information about complications during pregnancy and childbirth were not significantly associated with women's preference for home birth (Figures 7 and 8). The results of the study showed that there was a significant correlation between women's education and women's preference for home birth. Women with higher education were 53% less likely to prefer home birth than women with lower education (Figure 5). The study also showed that there was a significant relationship between women's age and preference for home birth. The likelihood of home birth was 78% higher in the younger age group than in the comparison group (Figure 6).

### 3.6. Outcome

The first interesting outcome of this systematic review and meta-analysis was the estimate of the pooled prevalence of women's preference for home birth in Ethiopia. Furthermore, the pooled prevalence of women's home delivery preference in Ethiopia was calculated and found that 2 out of 5 women preferred home delivery in Ethiopia. The second finding of interest is factors related to women's preference for home delivery. The result revealed that younger and less educated women are more likely to prefer home delivery.

## 4. Discussion

The result of this review showed that the estimated pooled prevalence of women's home birth preference in Ethiopia was 41.48% (95% CI (49.99; 63.56); *I*^2^ = 98.7%, *p* ≤ 0.001). Compared to studies conducted in Nigeria (15%) [20], Eritrea (11.7%) [21], Ghana (24.7%) [17], and Liberia (22%) [16], the prevalence of preference for home birth in the study was higher. The possible explanation could be based on differences in sample size, quality of health services, geographic area, sociodemographic and cultural background, implementation of strategies and policies, health facility availability, health facility infrastructure, and maternal perceptions and attitudes towards delivery. A study conducted in Uganda [35] found that 60% of study participants preferred home birth, which is higher than the results of this review. This discrepancy could be related to differences in the study setting and methods used to conduct the study.

The review found that women's educational status was significantly related to the choice of giving birth at home. Women with lower education are less likely to use health services from health institutions than women with higher education [12, 36]. Studies have shown that educated women prefer homebirths rather than uneducated women [37, 38]. The results of various studies support this conclusion [20, 28, 29]. Consequently, women's education plays an essential role in improving women's overall status and in helping them to make good decisions about their health [22].

The maternal age, especially in the youngest age group, is statistically significant for women's home birth preferences. Previous studies have shown that young women prefer homebirths over older age groups [39]. This could be due to a lack of experience in the labor and delivery process and complications compared to the older groups [28]. The young age group has less experience of the labor and delivery process and the complications that come with it. Therefore, young people do not have further information on the risk of home delivery and prefer home delivery rather than institutional delivery [17]. Furthermore, their financial dependence and expectations of natural and normal birth may limit their ability and the need for institutional delivery. Studies done in different regions of the country agree with the conclusions of this study [16, 17, 27, 40, 41]. This review has certain limitations: It included only cross-sectional studies that did not assess cause-and-effect analysis; Bias might exist because the search was in English only. The primary studies in this review did not include all 10 regions, and the majority of included studies relate to specific regions of the country, which can make it difficult to generalize results for all regions in Ethiopia.

## 5. Conclusion

According to this systematic review and meta-analysis, we concluded that two-fifths of Ethiopian women preferred home birth to institutional delivery. In addition, the finding found that young and uneducated women were more likely to prefer home delivery to institutional delivery. Therefore, all stakeholders and policy makers are strongly encouraged to take immediate actions. We also recommend that the Ethiopian government, particularly the Ministry of Health, must monitor and assess the status of home delivery in each region with the high prevalence of preference for home birth, especially in the Oromia region. Health workers need to provide health education for young age groups and women with low levels of education. Finally, raising women's awareness at community and health facility levels is strongly recommended.

### 5.1. Implication of the Review

The fact is that home birth increases the risk of maternal and neonatal mortality and morbidity. To solve this problem, strategies need to be developed that require a national scientific study. The study provided data on the choice of home birth in Ethiopia and the factors associated with it. As the study shows, 41% of Ethiopian women choose home birth compared to other groups. This indicates there is a higher mortality and morbidity for women and their newborns. The review also has important implications for the achievement of the Sustainable Development Goals. Finally, the factors identified in this review need to be taken into particular consideration as they are accelerating the emergence of home delivery preferences. We, therefore, encourage all stakeholders to take immediate steps to maintain institutional delivery over home delivery by developing strategies and guidelines.

## Figures and Tables

**Table 1:** Critical appraisal results of eligible studies in this study on women's preference of home birth among delivered mothers in Ethiopia, 2023 (*n* = 14).

**Name of author**	**Q1**	**Q2**	**Q3**	**Q4**	**Q5**	**Q6**	**Q7**	**Q8**	**Q9**	**Total**
Fira A. et al. [28]	Y	Y	Y	Y	Y	Y	Y	Y	Y	9
Alemu SS et al. [29]	Y	Y	Y	N	Y	Y	Y	Y	Y	8
Abebe A. et al. [31]	Y	Y	Y	Y	Y	Y	N	Y	Y	8
Tsegaye et al. [30]	Y	Y	Y	Y	Y	Y	Y	Y	Y	9
Nigatu AM et al. [25]	Y	Y	Y	Y	Y	Y	Y	Y	Y	9
Mekie M et al. [26]	Y	Y	Y	Y	Y	Y	Y	Y	Y	9
Belay AS et al. [19]	Y	Y	Y	Y	Y	Y	Y	Y	Y	9
Bayu H et al. [24]	Y	Y	Y	U	Y	Y	Y	Y	Y	8
Yegezu B et al. [34]	Y	Y	Y	Y	Y	Y	Y	Y	Y	9
Haymanot.M et al. [22]	Y	Y	Y	Y	Y	Y	Y	Y	N	8
Henna.U et al. [32]	Y	Y	Y	Y	Y	Y	Y	Y	Y	9
Mohamud.M et al. [27]	Y	Y	Y	Y	Y	Y	Y	Y	Y	9
Weldearegay.HG [23]	Y	Y	Y	Y	Y	Y	Y	Y	Y	9
Yibeltal.T et al. [33]	Y	Y	Y	Y	Y	Y	Y	Y	N	8

*Note:* JBI critical appraisal checklist for studies reporting prevalence data: Q1—Was the sample frame appropriate to address the target population? Q2—Were study participants sampled appropriately? Q3—Was the sample size adequate? Q4—Were the study subjects and the setting described in detail? Q5—Was the data analysis conducted with sufficient coverage of the identified sample? Q6—Were the valid methods used for the identification of the condition? Q7—Was the condition measured in a standard, reliable way for all participants? Q8—Was there appropriate statistical analysis? Q9—Was the response rate adequate, and if not, was the low response rate managed appropriately?

Abbreviations: Y, yes; N, no; U, unclear.

**Table 2:** Study characteristics included in the systematic review of home birth preference among women who gave birth in Ethiopia.

**Author/s**	**Year**	**Region**	**Study setting**	**Study design**	**Study population**	**Sample**	**Preference for home delivery**
Fira et al.	2016	SNNPR	Bench Maji	CS	Delivered women	503	12%
Alemu et al.	2022	SNNPR	Arbaminch	CS	Delivered women	416	24%
Abebe et al.	2019	SNNPR	Gedeo	CS	Delivered women	772	25.7%
Tsegaye et al.	2019	Amhara	Debre-tabor	CS	Delivered women	394	29.2%
Nigatu et al.	2019	Amhara	Northwest	CS	Delivered women	528	52.7%
Mekie et al.	2019	Amhara	Simada	CS	Delivered women	346	56.4%
Belay et al.	2016	Amhara	Nortwest	CS	Delivered women	361	61.8%
Bayu et al.	2015	Amhara	Debre-markos	CS	Delivered women	393	19.6%
Yegezu et al.	2014	Oromia	Jimma	CS	Delivered women	277	64.6%
Haymanot et al.	2013	Oromia	Haramaya	CS	Delivered women	458	58.7%
Henna et al.	2020	Sidama	Hawassa	CS	Delivered women	402	38.8%
Mohamud et al.	2020	Somali	Jigjiga	CS	Delivered women	422	32%
Weldearegay	2013	Tigray	Ahferom	CS	Delivered women	458	58.7%
Yibeltal et al.	2015	Addis Ababa	Addis Ababa	CS	Delivered women	901	47.4%

Abbreviation: CS, cross-sectional.

**Table 3:** Sensitivity analysis of systematic review and meta-analysis on the women's preference for home birth and associated factors in Ethiopia.

**Study omitted**	**Estimate**	**(95% CI)**	
Fira et al. (2016)| [28]	37.994831	11.780307	122.5441
Alemu et al. (2022) [29]	32.599186	10.632115	99.952553
Abebe et al. (2019) [31]	32.897797	10.373701	104.32776
Tsegaye et al. (2019) [30]	32.089066	10.537155	97.721649
Nigatu et al. (2019) [25]	30.830765	10.085193	94.250656
Mekie et al. (2019) [26]	31.111948	10.307199	93.910416
Belay et al. (2016) [19]	30.901834	10.215609	93.476883
Bayu et al. (2015) [24]	33.216827	10.791134	102.24669
Yegezu et al. (2014) [34]	31.064867	10.326857	93.448181
Haymanot et al. (2013) [22]	30.750347	10.100573	93.616859
Henna et al. (2020) [32]	31.577421	10.408692	95.798149
Mohamud et al. (2020) [27]	31.908697	10.471609	97.230988
Weldearegay (2013) [23]	30.750347	10.100573	93.616859
Yibeltal et al. (2015) [33]	30.392796	9.6655331	95.568657
Combined	31.914075	10.82862	94.057058

## Data Availability

The datasets generated and/or analysed during the current study are not publicly available to prevent any kind of misuse by the public before publication but are available from the corresponding author upon reasonable request.

## Nomenclature

CI: confidence interval
OR: odds ratio
PICO: population comparison and intervention outcome
PRISMA: Preferred Reporting Items for Systematic Review and Meta-Analysis
PROSPERO: International Prospective Register of Systematic Review and Meta-analysis
SNNPR: Southern Nations, Nationalities, and People's Region
